# REDUCING LONELINESS AMONG OLDER ADULTS WITH ANIMATRONIC PETS

**DOI:** 10.1093/geroni/igz038.661

**Published:** 2019-11-08

**Authors:** Rifky Tkatch, Lizi Wu, Laurie Albright, James Murphy, James Schaeffer, Ellen Wicker, Charlotte S Yeh

**Affiliations:** 1 Optum, Ann Arbor, Michigan, United States; 2 UnitedHealthcare, Minneapolis, Minnesota, United States; 3 AARP Services, Inc., Washington, District of Columbia, United States

## Abstract

Pet ownership has been examined as a solution for loneliness. However, multiple challenges of pet ownership exist for older adults. Therefore, research efforts are considering the use of animatronic pets to reduce loneliness. The purpose of this study was to determine if ownership of animatronic pets would decrease loneliness and improve well-being among lonely older adults. Individuals were identified as lonely through a prior survey. Participants were provided with their choice of either an animatronic cat or dog and completed T1, T2, and T3 surveys. Response rates were high; 167 (63%) completed T1 and T2, and 125 (48%) also completed T3. T2 data indicated that loneliness decreased, while mental well-being, resilience, purpose in life, and optimism improved. At T3 mental well-being and purpose and life continued to improve. Animatronic pets appear to provide significant benefits for the well-being of lonely older adults.

